# *ARID1A* and *CTNNB1*/β-Catenin Molecular Status Affects the Clinicopathologic Features and Prognosis of Endometrial Carcinoma: Implications for an Improved Surrogate Molecular Classification

**DOI:** 10.3390/cancers13050950

**Published:** 2021-02-25

**Authors:** Antonio De Leo, Dario de Biase, Jacopo Lenzi, Giovanna Barbero, Daniela Turchetti, Marco Grillini, Gloria Ravegnini, Sabrina Angelini, Claudio Zamagni, Sara Coluccelli, Giulia Dondi, Pierandrea De Iaco, Anna Myriam Perrone, Giovanni Tallini, Donatella Santini, Claudio Ceccarelli

**Affiliations:** 1 Department of Experimental, Diagnostic and Specialty Medicine, Alma Mater Studiorum—University of Bologna, Via Massarenti 9, 40138 Bologna, Italy; giovanni.tallini@unibo.it (G.T.); claudio.ceccarelli@unibo.it (C.C.); 2 Molecular Pathology Laboratory, IRCCS Azienda Ospedaliero—Universitaria di Bologna/Azienda USL di Bologna, 40138 Bologna, Italy; dario.debiase@unibo.it; 3 Centro di Studio e Ricerca delle Neoplasie Ginecologiche, Alma Mater Studiorum—University of Bologna, 40138 Bologna, Italy; giovanna.barbero2@unibo.it (G.B.); daniela.turchetti@unibo.it (D.T.); gloria.ravegnini2@unibo.it (G.R.); s.angelini@unibo.it (S.A.); sara.coluccelli2@unibo.it (S.C.); giulia.dondi@aosp.bo.it (G.D.); pierandrea.deiaco@unibo.it (P.D.I.); myriam.perrone@aosp.bo.it (A.M.P.); donatella.santini@aosp.bo.it (D.S.); 4 Department of Pharmacy and Biotechnology, Alma Mater Studiorum—University of Bologna, 40126 Bologna, Italy; 5 Department of Biomedical and Neuromotor Sciences, Alma Mater Studiorum—University of Bologna, Via San Giacomo 12, 40126 Bologna, Italy; jacopo.lenzi2@unibo.it; 6 Unit of Medical Genetics, IRCCS Azienda Ospedaliero—Universitaria di Bologna, Via Massarenti 9, 40138 Bologna, Italy; 7 Pathology Unit, IRCCS Azienda Ospedaliero—Universitaria di Bologna, Via Massarenti 9, 40138 Bologna, Italy; marco.grillini@aosp.bo.it; 8 IRCCS Azienda Ospedaliero-Universitaria di Bologna, Via Albertoni 15, 40138 Bologna, Italy; claudio.zamagni@aosp.bo.it; 9 Division of Gynecologic Oncology, IRCCS Azienda Ospedaliero—Universitaria di Bologna, Via Massarenti 9, 40138 Bologna, Italy; 10 Centre for Applied Biomedical Research, Alma Mater Studiorum-University of Bologna, 40138 Bologna, Italy

**Keywords:** endometrial cancer, molecular classification, *ARID1A*, *CTNNB1*/β-catenin, prognosis, high-risk endometrial cancer

## Abstract

**Simple Summary:**

Translation of the molecular characterization of endometrial cancer into the clinical practice is emerging as a challenge. This study investigates the feasibility and the prognostic impact of the novel surrogate TCGA molecular classification of endometrial carcinoma into the clinical setting proposing an immuno-molecular algorithm supplemented with *ARID1A* and *CTNNB1*/β-catenin analysis. The integrated clinicopathologic and molecular approach developed in this study could represent a workable and useful method in routine clinical practice for improving risk stratification and patient management.

**Abstract:**

The collaborative Cancer Genome Atlas (TCGA) project identified four distinct prognostic groups of endometrial carcinoma (EC) based on molecular alterations: (i) the ultramutated subtype that encompasses *POLE* mutated (*POLE*) cases; (ii) the hypermutated subtype, characterized by MisMatch Repair deficiency (MMRd); (iii) the copy-number high subtype, with p53 abnormal/mutated features (p53abn); (iv) the copy-number low subtype, known as No Specific Molecular Profile (NSMP). Although the prognostic value of TCGA molecular classification, NSMP carcinomas present a wide variability in molecular alterations and biological aggressiveness. This study aims to investigate the impact of *ARID1A* and *CTNNB1*/β-catenin alterations by targeted Next-generation sequencing (NGS) and immunohistochemistry (IHC) in a consecutive series of 125 molecularly classified ECs. NGS and IHC were used to assign surrogate TCGA groups and to identify molecular alterations of multiple target genes including *POLE*, *PTEN*, *ARID1A*, *CTNNB1*, *TP53*. Associations with clinicopathologic parameters, molecular subtypes, and outcomes identified NSMP category as the most heterogeneous group in terms of clinicopathologic features and outcome. Integration of surrogate TCGA molecular classification with *ARID1A* and β-catenin analysis showed NSMP cases with *ARID1A* mutation characterized by the worst outcome with early recurrence, while NSMP tumors with *ARID1A* wild-type and β-catenin alteration had indolent clinicopathologic features and no recurrence. This study indicates how the identification of *ARID1A* and β-catenin alterations in EC represents a simple and effective way to characterize NSMP tumor aggressiveness and metastatic potential.

## 1. Introduction

Endometrial cancer (EC) is the most common gynecological cancer, with annual incidence rates in Western countries ranging between 15 and 25 per 100.000 women [1,2]. About 15–20% of patients with endometrial cancer have high-risk disease and follow an aggressive clinical course. EC prognosis is traditionally defined by a combination of clinical and histopathologic (e.g., histotype, grade, lymphovascular invasion, stage) criteria that are also used to tailor surgery and to select patients for adjuvant therapy. Unfortunately, the assessment of histologic parameters is poorly reproducible and conventional clinicopathologic features do not reliably predict either the patient’s response to the available treatment or the definition of personalized forms of therapy [3]. The Cancer Genome Atlas (TCGA) endometrial collaborative project identified four distinct prognostic EC groups based on molecular alterations: (i) the ultramutated subtype that encompassed *POLE* exonuclease domain mutated (*POLE*) cases (excellent prognosis); (ii) the hypermutated subtype, characterized by MisMatch Repair deficiency (MMRd) (intermediate prognosis); (iii) the copy-number high subtype, with p53 abnormal/mutated features (p53abn) (poor prognosis); (iv) the copy-number low subtype, also known as No Specific Molecular Profile-NSMP (intermediate prognosis) [4]. Translation of the TCGA molecular groups into the clinical practice is an emerging challenge. Two groups independently proposed and validated the same surrogate markers (*POLE* mutation, microsatellite instability, and p53 mutation/alteration) to identify TCGA groups in routine clinical practice [5,6,7]. The ProactiveMolecular Risk Classifier for Endometrial Cancer (ProMisE) algorithm applies *POLE* mutation, p53 and MMR protein expression analyses to sequentially assign first the MMR deficient group, then *POLE* mutant, and finally aberrant p53 cases; the remaining tumors are categorized as p53 normal [6,7]. Similarly, the TransPORTEC initiative has identified four prognostic groups, identifying first *POLE* proofreading mutant tumors, then subsequently MMRd tumors, p53-mutant tumors, and a group with no specific molecular profile (NSMP) [5]. In both algorithms, prognostic signatures emerged by stratifying endometrial cancer tumors according to these specific molecular criteria.

Although the four TCGA molecular groups appear to have different prognosis, it has become clear that the NSMP tumors (which account for the majority of endometrial cancer cases) represent a heterogeneous group of carcinomas with variable molecular alterations and divergent clinical outcomes. The NSMP group predominantly consists of low-grade endometrioid-type endometrial carcinomas characterized by alterations in PI3K/AKT and WNT/β-catenin signaling pathways with relatively frequent mutations in exon 3 of *CTNNB1* (52%) [4]. Some studies evaluated the potential prognostic impact of *CTNNB1* mutations in low-grade early-stage EC showing a significantly worse recurrence-free survival [8,9]. The prognostic significance of *CTNNB1* mutations within NSMP EC may qualify *CTNNB1* mutant EC as a separate biomolecular entity representing the fifth molecular EC group (after exclusion of cases belonging to the *POLE*, MMRd and p53 abn classes).

Among the molecular alterations investigated in EC stand out those affecting proteins of the switch/sucrose non-fermenting chromatin remodeling complex (SWI/SNF), in particular SMARCA4 (BRG-1), SMARCB1 (INI-1) and ARID1A/B. The SWI/SNF complex performs essential functions that permit gene expression, altering the structure of nucleosomes and allowing direct access to genes for their transcription. Mutations affecting the subunits that make up this complex may result in the alteration of several chromatin-related processes, including DNA repair, DNA synthesis, mitosis, and genomic instability. The *AT-rich interactive domain 1A* (*ARID1A*) gene encodes the protein BAF250a, which is a key component of the multi-protein SWI/SNF chromatin remodeling complex [10]. Mutations in *ARID1A* occur across the entire gene and are generally inactivating (frameshift or truncation). These mutations result in loss of ARID1A protein, which is detectable by immunohistochemistry, consistent with a loss of function mechanism of oncogenesis. Loss of ARID1A expression determines a complex set of SWI/SNF functional alterations: (i) defects in the enhancer-mediated gene regulation of cell cycle checkpoint activation in response to DNA damage [11]; (ii) alteration in the expression of genes regulated by nuclear hormonal receptors; and (iii) a deregulation of the developmental gene expression while maintaining cell self-renewal, survival and proliferative capacity [12].

*ARID1A* is mutated in approximately 30–40% of both low- and high-grade endometrioid ECs, but not in serous endometrial carcinomas [13,14,15]. Some studies demonstrated that *ARID1A* mutation is associated with mismatch repair deficiency and normal p53 expression [16,17]. Furthermore, loss of ARID1A in complex atypical hyperplasia is associated with malignant transformation and concurrent EC [18], and promotes epithelial-to-mesenchymal transition (EMT) and myometrial invasion [19].

All these observations about the role of ARID1A in regulating enhancer-mediated gene expression, in tumor suppression and in the regulation of differentiation programs, have encouraged us to investigate the significance of ARID1A alterations in refining the molecular classification of EC.

The aims of this study are: (I) to evaluate the prognostic stratification based on conventional clinicopathologic criteria according to ESMO 2016 risk group criteria [20] in a single center, population-based series of EC; (II) to investigate the feasibility and the prognostic impact of the novel surrogate TCGA molecular classification of endometrial carcinoma; (III) to correlate EC histopathologic characteristics with molecular subtypes; (IV) to define the relevance of *ARID1A* and *CTNNB1* mutations and their predictive-prognostic weight with particular reference to the NSMP group; (V) to integrate ESMO 2016 risk stratification criteria with molecular subtyping; (VI) to propose a workable and useful algorithm into the routine clinical practice for improving risk stratification and patient management. 

## 2. Materials and Methods

### 2.1. Study Cohort and Clinicopathologic Data

The study was approved by the local research ethics committee CE-AVEC (Comitato Etico—Area Vasta Emilia Centro, registration n. 27/2019/Sper/AOUBo). All patients signed an informed consent permitting the use of their normal as well as neoplastic tissue and the data necessary for the study. All patients underwent surgical resection with staging at the Division of Gynecologic Oncology of “IRCCS Azienda Ospedaliero—Universitaria di Bologna” (Bologna, Italy) [21]. Formalin-fixed paraffin-embedded (FFPE) tissue blocks containing representative tumor samples were selected from 125 consecutive primary endometrial carcinomas in the files of the Pathology Unit of “IRCCS Azienda Ospedaliero—Universitaria di Bologna” (Bologna, Italy). The selected blocks were used to assess histopathologic parameters, for immunohistochemical and molecular analyses. In order to minimize biases due to tumor heterogeneity we used whole tissue sections from surgical resection rather than tissue microarrays or small biopsy samples for histologic, immunohistochemical and molecular analyses. The histology slides and all histopathologic parameters were thoroughly reviewed by two expert pathologists (D.S., A.D.L.). Clinical data were obtained from clinical, surgical and pathologic records reported in a comprehensive clinicopathologic database included: age at diagnosis, Body Mass Index (BMI), International Federation of Gynecology and Obstetrics (FIGO) stage determined using surgical reports, ESMO 2016 risk stratification group, type of surgery, peri-operative complications, imaging studies, pathology reports, and clinical findings including follow-up data.

- Tumors were classified according to standard histopathologic criteria following the World Health Organization classification of tumors [22] and graded using standard FIGO criteria [23].
- The depth of myometrial invasion was recorded in all cases as a percentage of myometrial thickness.
- The pattern of myometrial invasion was reported, specifying whether microcystic, elongated and fragmented (MELF) [24] and/or as single invasive cells or small groups of cells (tumor budding) [25]. Characteristics of the MELF pattern include the presence of invasive small dilated glands lined by cuboidal or flattened cells with eosinophilic cytoplasm and with slit-like appearance. This invasive pattern typically has a myxoid to granulation-like reaction in the surrounding myometrium. Tumor budding is defined as invasive single/small group of cells without formation of defined structures frequently lying in an edematous or myxoid background.
- Lymphovascular space invasion (LVSI) is defined by the presence of tumor fragments within endothelial-lined vascular/lymphatic spaces outside the immediate invasive border. Intratumoral LVSI foci were not considered. A semi-quantitative *three- tiered* scoring system was applied: no LVSI; focal (a single focus of LVSI recognized around the tumor); substantial (diffuse or multifocal LVSI around the tumor) [26,27].
- The presence of extensive tumor necrosis was reported; necrosis only within glands or at the tumor’s surface was not scored.
- Tumor heterogeneity were reported when a tumor had two or more clearly separate morphological patterns, and each constituting at least 10% of the tumor [28].
- Tumor infiltrating lymphocytes were assessed considering intraepithelial tumor infiltrating lymphocytes (iTILs; lymphocytes located within the tumor epithelium) and stromal tumor infiltrating lymphocytes (sTILs; lymphocytes in the stroma immediately adjacent to the tumor epithelium). The number of intraepithelial lymphocytes was counted in 10 high-power fields (HPF, ×400 magnification) with the highest density of TILs. The cut-off of 40 lymphocytes per 10 HPF was used to define a high iTIL score [28,29]. sTILs counting was evaluated at ×400 magnification field from the invasive border and performed according to the semi-quantitative method of Shia: sTILs absent/mild and sTILs moderate/high [28,29].
- The mitotic index was expressed as the number of mitoses per 10 high-power fields (×400 magnification).

### 2.2. Immunohistochemistry

The details of the immunohistochemistry (IHC) methods to assess p53, PTEN, ARID1A, β-catenin, MLH1, PMS2, MSH2, MSH6, and Ki67 are described in supplementary Table S1. Slides were evaluated by two observers (ADL, CC) without knowledge of the patient’s characteristics and outcome.

#### 2.2.1. Immunohistochemical Assessment and Evaluation of p53 Expression

p53 was considered abnormal/mutant-like (p53abn) if more than 50% of the tumor cells showed strong positive nuclear staining, or when areas (subclones) consisting of > or =50% positive tumor cells were present, or when no nuclear p53 staining was evident in the entire tumor. In addition to nuclear overexpression of p53 and complete absence of nuclear p53 staining (null pattern) a third mutant pattern showing strong cytoplasmic overexpression has been considered [30].

#### 2.2.2. Immunohistochemical Assessment and Evaluation of PTEN Expression

PTEN was considered negative if no cytoplasmic/nuclear immunostain was identified in the neoplastic cells; cases were considered positive if uniform or heterogeneous staining was identified in the neoplastic cells [31].

#### 2.2.3. Immunohistochemical Assessment and Evaluation of ARID1A Expression

ARID1A nuclear staining was scored as follows: negative “loss of expression”, “positive” (weak or strong) or as “clonal loss” [17]. In the final analysis, “clonal loss” was reclassified as “loss of expression” as this pattern corresponded to subclonal *ARID1A* mutations. 

#### 2.2.4. Immunohistochemical Assessment and Evaluation of β-Catenin Expression

β-Catenin was classified as “normal” when only membranous/cytoplasmic staining was present or “abnormal” when there was nuclear immunoreactivity. Weak nuclear staining associated with cytoplasmic/membranous expression in occasional cells was considered “normal” because the same pattern of immunoreactivity was observed in normal endometrium [32,33].

#### 2.2.5. Immunohistochemical Assessment and Evaluation of MMR Protein Expression

MLH1, PMS2, MSH2, and MSH6 were scored negative if no nuclear immunostaining was present. Cases were considered mismatch repair deficient (MMRd) if one of the four proteins was absent or if MLH1/PMS2 or MSH2/MSH6 were negative [34].

#### 2.2.6. Immunohistochemical Assessment and Evaluation of Ki67 Proliferative Index

The evaluation of the proliferative index (Ki67) in the neoplastic population was carried out quantitatively using image analysis with the Image-Pro Plus 5.1 software (Media Cybernetics Inc., Silver Spring, MD, USA) in at least forty ×200 magnification fields, and expressed as the ratio (%) between the positive neoplastic cells and the total neoplastic cells.

### 2.3. DNA Extraction and Next Generation Sequencing

DNA was extracted from FFPE tissue starting from two to four 10-µm-thick sections, according to the amount of neoplastic tissue present in the paraffin block. The areas of interest were marked on the control hematoxylin and eosin (H&E) stained slide and manually dissected under microscopic guidance using a sterile blade. DNA was extracted using the Quick Extract Kit (Epicentre, Madison, WI, USA) and quantified using the “Qubit” fluorometer (ThermoFisher Scientific, Waltham, MA, USA). Samples were analyzed using a customized panel of genomic regions and sequenced using the Gene Studio S5 sequencer (ThermoFisher Scientific), according to the manufacturer’s instruction (ThermoFisher Scientific) as previously published [35]. Template preparation was performed using the Chef Machine instrument (ThermoFisher Scientific) and then sequenced using an Ion 530 chip. The panel included a total of 169 amplicons within the following gene regions: *ARID1A* (all CDS region), *BRAF* (exon 15), *cKIT* (exons 8, 9, 11, 13, 17), *CTNNB1* (exons 3, 7, 8), *HRAS* (exons 2–4), *KRAS* (exons 2–4), *NRAS* (exons 2–4), *PIK3CA* (exons 10, 21), *POLE* (exons 9–14), and *TP53* (exons 4–9). 

Only nucleotide variations in at least 5% of the total number of reads analyzed were considered for mutational call. The sequences obtained were analyzed using the Ion Reporter Software (version 5.10.5, ThermoFisher Scientific) and the Integrative Genomics Viewer 2.5 (IGV) tool (Available online: http://software.broadinstitute.org/software/igv/ (accessed on 15 December 2020).

### 2.4. Methylation Specific PCR

Tumors with loss of MLH1 protein expression were selected for further testing for methylation status of the 5’ regulatory region of MLH1, using methylation-specific PCR, with previously reported primers [36].

### 2.5. Assignation of Carcinomas to Surrogate TCGA Molecular Groups and NSMP Subgroups

**Figure 1:**
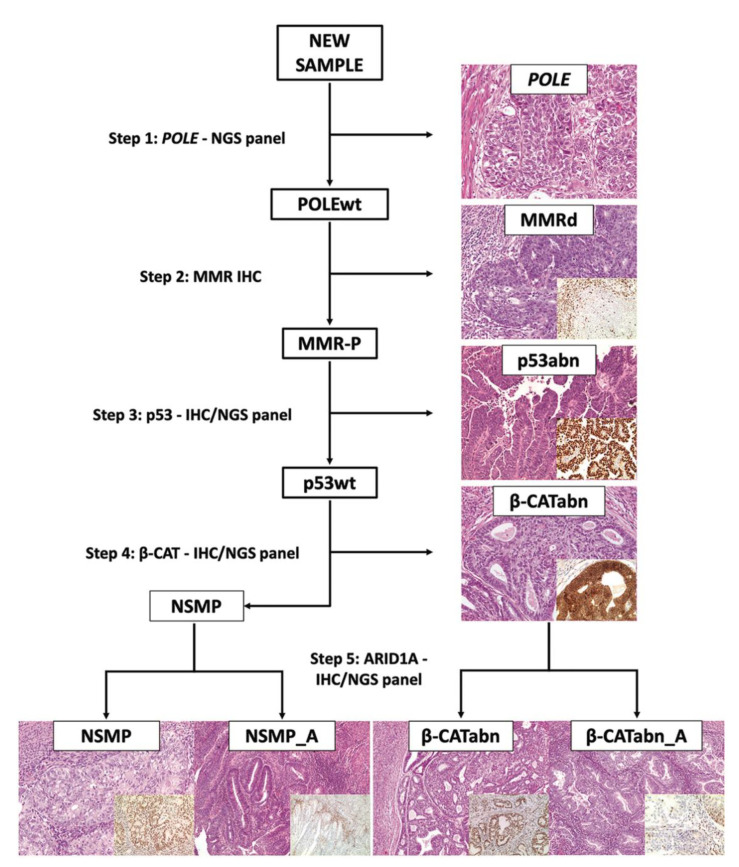
Diagnostic algorithm for the “immuno-molecular” classification of endometrial carcinoma.

The steps in immuno-molecular classification are illustrated in Figure 1. First, all cases were assessed for pathogenic *POLE* mutations to identify “ultramutated” group tumors (*POLE*). Diagnostic interpretation of *POLE* mutations was based according to reported guidelines [37]. The next assessment was the immunohistochemical determination of mismatch repair (MMR) proteins expression to identify MMR deficient (MMRd) tumors and to assign tumors to the TCGA “hypermutated” group (in absence of *POLE* mutation). Subsequently, tumors were evaluated by IHC for p53 to detect p53 abnormal (p53abn) tumors corresponding to the “copy-number high/serous-like” TCGA group. Tumors exhibiting normal p53 and MMR expression by IHC with no *POLE* mutations, were defined as “No specific molecular profile” (NSMP) tumors and correspond to the “copy-number low” subgroup in the TCGA. This latter group was split into two subgroups according to *CTNNB1* mutations/β-catenin abnormal expression: β-catenin abnormal (β-CATabn) and β-catenin wild type (NSMP). Each of these subgroups was further stratified according to *ARID1A* mutations/ARID1A loss of expression (β-CATabn/β-CATabn_A, NSMP/NSMP_A).

### 2.6. Statistics

Summary statistics are reported as numbers (percentages) or mean ± standard deviation. Crude comparisons between groups were performed using Fisher’s exact test, chi-squared test, Kruskal–Wallis test, t-test and Mann–Whitney test, when appropriate. We used the Kaplan-Meier estimator to display disease-free survival and overall survival following surgery; the equality of survivor functions was assessed using the log-rank test. All deaths from disease were considered an event; all recurrences (local, regional, and distant) were considered as an event. All analyses were carried out using Stata software, version 15 (Stata Statistical Software: Release 15, 2017; StataCorp LLP, College Station, TX, USA). The significance level was set at 5%.

## 3. Results

### 3.1. Clinicopathologic Features of Endometrial Carcinoma and Conventional Prognostic Stratification

Clinicopathologic characteristics of the 125 patients are shown in Table 1. Median patient age at diagnosis was median 62.7 years. The median body mass index (BMI; kg/m^2^) was 27.5. Histologic classification includes 90 (72%) endometrioid, 17 (13.6%) dedifferentiated/undifferentiated, 15 (12.0%) serous, and 3 (2.4%) clear cell endometrial carcinomas. Grade distribution is homogeneous and includes 36 (28.8%) grade 1, 35 (28.0%) grade 2 and 54 (43.2%) grade 3. Lymph node metastases are present in 24 (19.2%) patients. 

**Figure 2:**
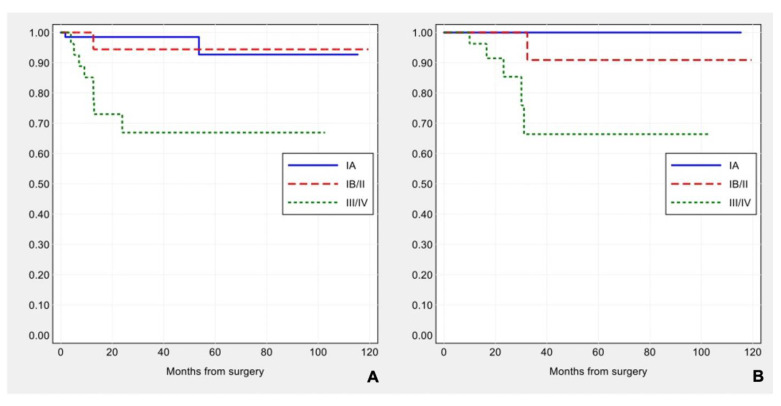
Kaplan–Meier estimates of disease-free survival (**A**) and overall survival (**B**) by FIGO stage.

Applying FIGO stage/AJCC 8th ed., 71 (56.8%) patients were stage IA, 18 (14.4%) stage IB, 4 (3.2%) stage II, 30 (24.0%) stage III and 2 (1.6%) stage IV. Median follow-up was 19.1 months (range 1–119.5). Eleven (8.8%) patients developed disease progression during follow-up (one local and 10 distant recurrences), and six (4.8%) patients died of the disease. FIGO stage was significantly associated with disease-free survival (log-rank: χ^2^ = 14.64, *p*-value < 0.001) and overall survival (log-rank: χ^2^ = 11.64, *p*-value = 0.003) (see Figure 2A,B).

**Figure 3:**
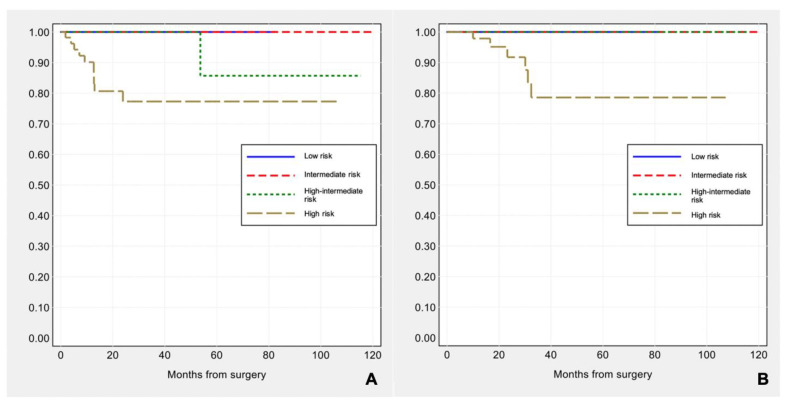
Kaplan–Meier estimates of disease-free survival (**A**) and overall survival (**B**) by ESMO risk groups.

Applying ESMO 2016 risk stratification criteria, 18 (14.4%) carcinomas were low risk, 8 (6.4%) intermediate risk, 42 (33.6%) high-intermediate and 57 (45.6%) high risk. ESMO 2016 risk groups were significantly correlated with disease-free survival (log-rank: χ^2^ = 9.47, *p*-value = 0.024), but not with overall survival (log-rank: χ^2^ = 5.63, *p*-value = 0.131) (see Figure 3A,B).

### 3.2. Molecular TCGA Group Assignment

Surrogate TCGA molecular typing of the 125 EC cases classified tumors into the following groups: 9 (7.2%) *POLE* group, 41 (32.8%) MMRd group, 26 (20.8%) p53abn group, 49 (39.2%) NSMP group. The association between TCGA molecular groups and clinicopathologic parameters (BMI, histotype, grade, FIGO stage, MELF, tumor budding, TILs, mitoses, Ki67 proliferative index) are shown in Table 2. Nine cases (7.2%) show more than one molecular feature (so-called “multiple classifier” tumors): 2 tumors are *POLE*-p53abn, 1 tumor is *POLE*-MMRd, 1 tumor is *POLE*-MMRd-p53abn, 5 tumors are MMRd-p53abn. The features of the EC cases according to the surrogate TCGA molecular classification are as follows.

#### 3.2.1. *POLE*-Mutated Tumors

**Figure 4:**
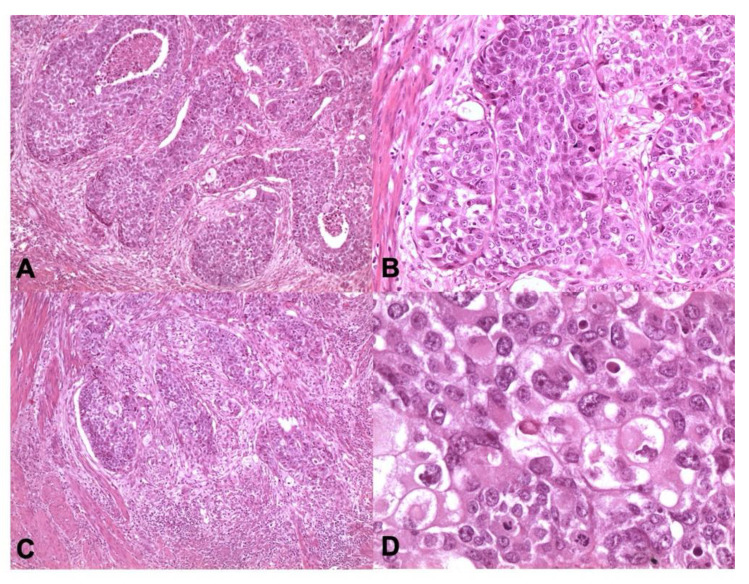
*POLE*-mutated endometrioid carcinomas. *POLE* carcinomas may have eosinophilic tumor cells with marked atypical nuclei and lymphoid infiltrate (**A**,**C** ×100 magnification, **B** ×200 magnification, **D** ×400 magnification; Hematoxylin and Eosin-H&E).

Predominantly endometrioid, grade 3 and morphologically heterogeneous in half of the cases, statistically associated with characteristic myometrial infiltration patterns (MELF and tumor budding), intense intra- and peri-tumoral lymphocytic infiltrate (iTILs and sTILs), high mitotic rate and high Ki67 proliferative index (see Figure 4). In the *POLE* group, lymph node metastases are present in one of nine cases (11.1%). 

#### 3.2.2. MMRd Tumors

**Figure 5:**
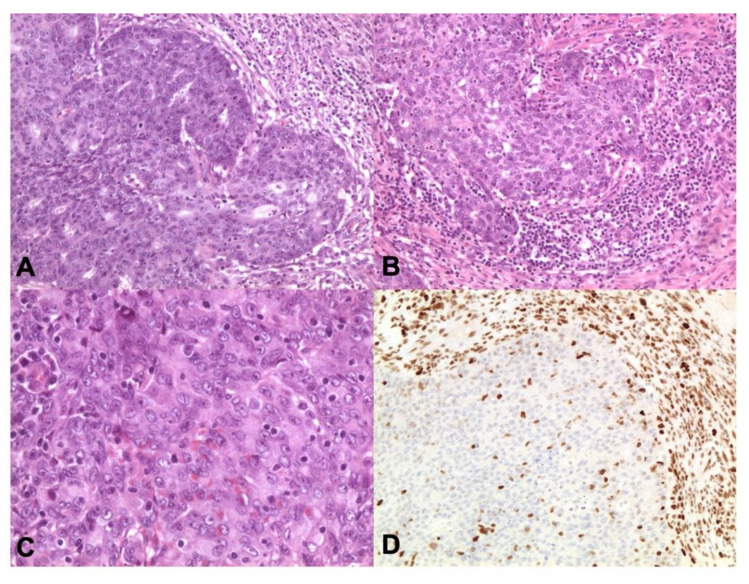
MMRd endometrioid carcinoma. The tumor shows numerous intra- and peritumoural TILs (**A**,**B** ×200 magnification, **C** ×400 magnification; Hematoxylin and Eosin-H&E) and diffuse immunohistochemical nuclear loss of MLH1 (**D** ×400 magnification).

Characterized by endometrioid or dedifferentiated/undifferentiated histotypes, homogeneous histologic grade distribution, association with MELF pattern of myometrial invasion, tumor budding and high iTILs /sTILs (see Figure 5). Lymph node metastases present in eight cases (19.5%).

#### 3.2.3. p53abn Tumors

**Figure 6:**
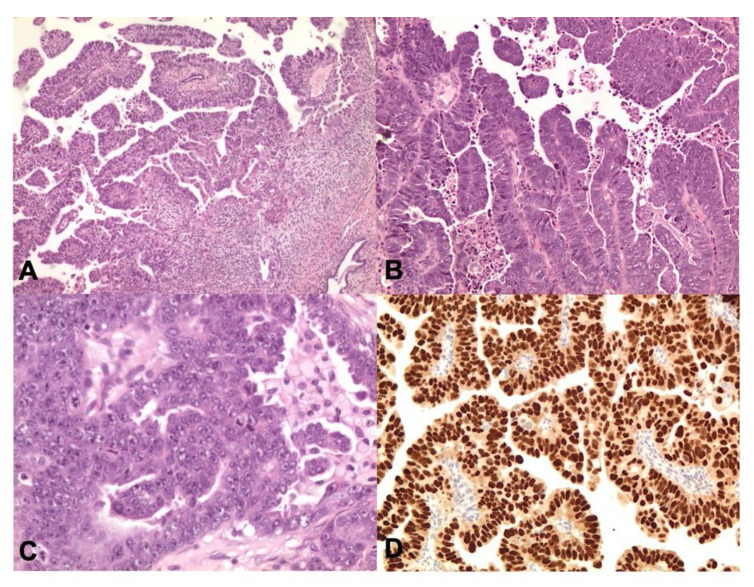
p53 abn carcinoma. Tumor have serous histotype with marked nuclear pleomorphism, macronucleoli, and conspicuous mitotic activity (**A** ×100 magnification, **B** ×200 magnification, **C** ×400 magnification; Hematoxylin and Eosin-H&E) and p53 abnormal/mutant-like expression (**D** ×200 magnification).

Significantly associated with serous histotype, grade 3, high mitotic rate and high Ki67 proliferative index (see Figure 6). Metastatic lymph nodes in 10 cases (38.5%).

#### 3.2.4. NSMP Tumors

**Figure 7:**
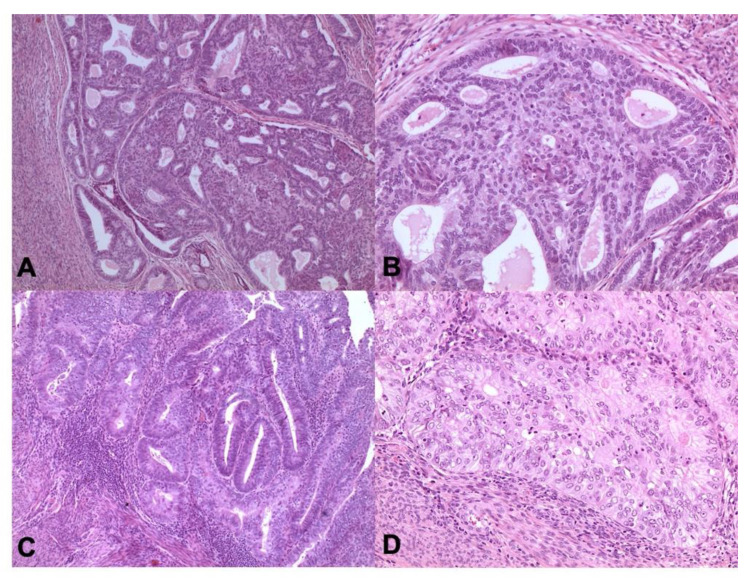
NSMP tumors are prevalently endometrioid, low grade morphology (**A,C** ×100 magnification, **B**,**D** ×200 magnification; Hematoxylin and Eosin-H&E).

Endometrioid, more frequently grade 1–2, lower mitotic activity and Ki67 proliferative index (compared with the other EC groups), with metastatic lymph node metastatic in five cases (10.5%) (see Figure 7).

**Figure 8:**
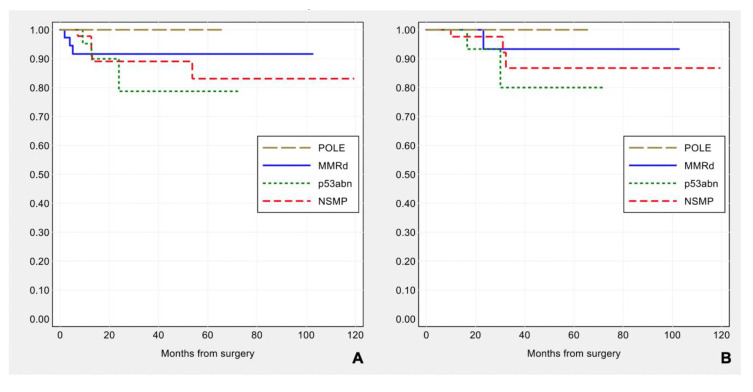
Kaplan–Meier estimates of disease-free survival (**A**) and overall survival (**B**) by surrogate TCGA molecular groups.

Prognostic impact of the surrogate TCGA molecular group classification: *POLE* tumors show the most favorable outcome, without any recurrence, while recurrent disease is observed in 3/41 (7.3%) MMRd, 3/26 (11.5%) p53abn, 5/49 (10.2%) NSMP subtypes. The patient outcome by molecular classification (Figure 8) is consistent with that previously reported [5,6,7,38,39,40,41], but in this series does not reach statistical significance for disease-free survival (log-rank: χ^2^ = 1.29, *p*-value = 0.730) and for overall survival (log-rank: χ^2^ = 1.98, *p*-value = 0.576).

Considering ESMO high-intermediate and high-risk groups, surrogate TCGA molecular classification was not statistically correlated with disease-free survival and overall survival (log-rank: χ^2^ = 1.45, *p*-value = 0.694 and χ^2^ = 2.19, *p*-value = 0.534, respectively) (see Figure S1).

### 3.3. CTNNB1 Mutations/β-Catenin Abnormal Expression

Of the 125 endometrial carcinomas examined, 21 (16.8%) tumors carry exon 3 *CTNNB1* mutations and concomitant nuclear expression of β-catenin. For the *CTNNB1* mutant ECs, nuclear localization of β-catenin in neoplastic cells ranges from 5% to 60% (mean 19.8%). Clinicopathologic features of endometrial carcinoma associated with *CTNNB1* mutations/nuclear expression of β-catenin are shown in Table S2. In summary, β-catenin mutated cases are characterized by young age at diagnosis, high BMI, low mitotic rate and Ki67 proliferative index, no tumor necrosis, low TILs counts, and prevalently encompass into the NSMP molecular group (16/21 cases).

Considering the prognostic role of *CTNNB1* mutations reported in the literature [8,9,33,42], and its association with the NSMP group, this latter was divided into two subgroups: 15/43 (34.9%) β-catenin abnormal (β-CATabn) cases, and 28/43 (65.1%) NSMP *CTNNB1* wild-type (NSMP) cases. By integrating the surrogate TCGA molecular groups with the β-CATabn subgroup, β-catenin mutated tumors are similar to those of the NSMP subtype, except for lower mitotic rate and Ki67 proliferative index (see Table 3).

### 3.4. ARID1A Mutations/ ARID1A Loss of Expression

IHC ARID1A loss is present in 69/125 (55.2%) and it is concordant with *ARID1A* mutations. The clinicopathologic features associated with *ARID1A* mutation are shown in Table S3. *ARID1A* alteration is significantly associated with endometrioid and dedifferentiated/undifferentiated histotypes, MMRd and *POLE* molecular subgroups, MELF pattern of invasion, high TILs and high ki67 proliferative index. 

Integrating *ARID1A* analysis in molecular subtyping, *ARID1A* alteration is found in 8/9 (88.9%) *POLE*, 33/41 (80.5%) MMRd, 3/26 (11.5%) p53abn, 19/33 (57.6%) NSMP and in 7/16 (43.8%) β-CATabn group tumors. Of note, ARID1A clonal loss (“clonal loss” IHC pattern) corresponding to subclonal inactivating *ARID1A* mutations is identified in 27/69 (39.1%) mutated tumors: 5 *POLE*, 11 MMRd, 2 p53abn, 5 NSMP, 4 β-CATabn. In the *POLE*, MMRd and p53abn groups, ARID1A loss/mutation is not associated with specific clinicopathologic features. 

In contrast, in the β -CATabn subgroup, loss/mutation of ARID1A is associated with older age (*p* = 0.044), high grade (*p* = 0.001), extensive necrosis (*p* = 0.019), tumor budding (*p* = 0.019), high sTILs and iTILs (*p* = 0.009 and *p* = 0.012, respectively), high mitotic rate and high Ki-67 proliferative index (*p* = 0.001 and *p* = 0.003, respectively). *ARID1A* alteration in NSMP (NSMP_A) group correlates with high Ki67 proliferative index and with tumor recurrence (see Table 4).

**Figure 9:**
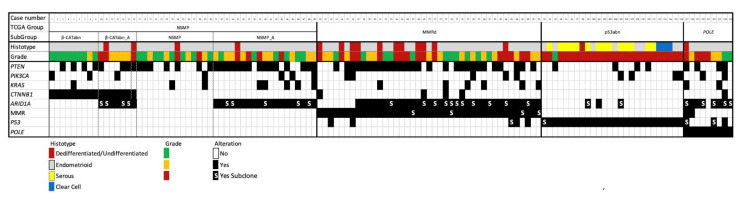
Immuno-molecular characterization of 125 endometrial carcinomas stratified according to histopathologic, immunophenotypic and molecular analyses.

The heatmap summarizes mutation status/IHC alterations in the different molecular groups (Figure 9).

### 3.5. Correlation of Immuno-Molecular Subgroups with Clinical Outcome

**Figure 10:**
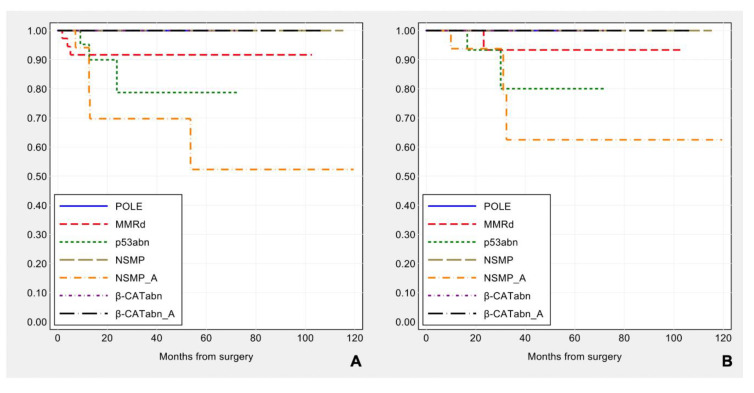
Kaplan–Meier estimates of disease-free survival (**A**) and overall survival (**B**) by molecular subgroup, including β-catenin and *ARID1A* alterations.

The prognostic impact of surrogate TCGA molecular classification integrated with *ARID1A* and *CTNNB1*/β-catenin analyses was evaluated and the molecular subgroups tended to be associated with different disease-free survival (log-rank: χ2 = 12.13, *p*-value = 0.059), but not with overall survival (log-rank: χ2 = 9.30, *p*-value = 0.157) (see Figure 10). 

**Figure 11:**
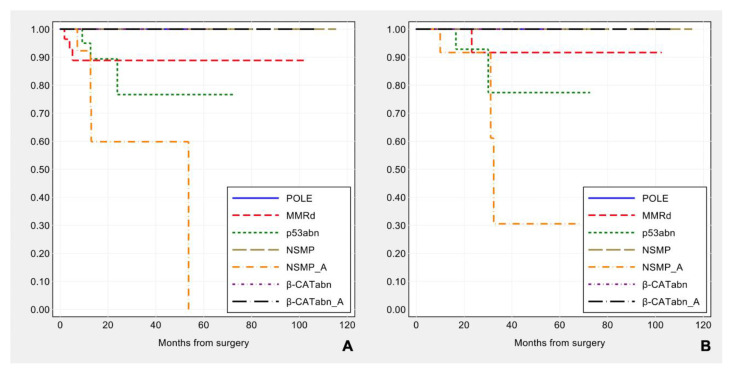
Kaplan–Meier estimates of disease-free survival (**A**) and overall survival (**B**) in patients at high-intermediate and high risk according to ESMO (n = 99), by molecular subgroup including β-catenin and *ARID1A* alterations.

Considering the disease-free survival, if we limit the analysis to ESMO high-intermediate and high risk groups, *POLE*, NSMP *ARID1A* wild-type, β-CATabn groups show a favorable prognosis, MMRd tumors have an intermediate outcome, while patients with either p53abn or NSMP with *ARID1A* mutation (NSMP_A) tumors feature a worse prognosis and are associated with a higher rate of recurrence (log-rank: χ2 = 14.07, *p*-value = 0.029). As regards the overall survival, the statistical significance is borderline (χ2 = 12.60, *p*-value = 0.050) (see Figure 11).

## 4. Discussion

In 2013, the multi-institutional TCGA project identified four distinct prognostic groups for the molecular classification of endometrial carcinoma. The TCGA study stratified EC into clinically low-risk (*POLE*-ultramutated), intermediate-risk (copy-number low/NSMP and hypermutated/MMRd groups) and high-risk (copy-number high/p53 mutant group) categories [4].

Subsequently, two studies (ProMisE and PORTEC) have developed and validated molecular classification tools based on widely accessible surrogate markers capable of discriminating four molecular EC subclasses with distinct prognostic outcomes, similar—but not identical—to those outlined in the TCGA study [5,6,7]. In contrast to the previous multitude of biomarkers reported in literature, these routine molecular classifiers provide biologically relevant information that is potentially useful for both research and clinical applications to better stratify ECs.

In spite of the prognostic value of the novel molecular classification, the so-called copy-number low/No Specific Molecular Profile (NSMP) group represents the majority of ECs with intra-class heterogeneity in terms of biological behavior and clinical outcomes. In the NSMP group the presence of chromosome 1q amplification, *CTNNB1* mutations, and L1CAM expression may predict an increased risk for recurrence. Activating mutations in exon 3 of *CTNNB1* are likely early drivers in endometrial carcinogenesis and are identified in a significant proportion (26–52%) of NSMP cases [4,8,9,33,42]. For these reasons, *CTNNB1*-mutated ECs may be regarded as a fifth molecular group.

In this study we investigated the feasibility of the surrogate TCGA molecular classification in our cohort of EC patients correlating it to conventional clinicopathologic characteristics. To analyze the prognostic heterogeneity of the NSMP tumors, we also aimed to explore the significance of *CTNNB1* and *ARID1A* alterations in this EC group by assessing their impact on disease recurrence and clinicopathologic characteristics.

A limitation of our study is the relatively low number of cases and the fact that the study is retrospective and from a single institution. However, meticulous histopathologic analysis and the use of whole tumor sections (as opposed to biopsy samples) for molecular and immunohistochemical analyses to avoid biases due to tumor heterogeneity insure the validity of our results.

Interestingly, in our EC series the FIGO stage has proved to be a robust parameter, being strongly correlated to both disease-free survival and overall survival. In addition, the integration of conventional clinicopathologic parameters according to ESMO 2016 criteria has allowed to divide our EC series into different risk groups statistically related to recurrence.

Applying surrogate TCGA classification, our data confirm the previously reported distribution of the four molecular groups of endometrial carcinoma [5–7, 38–41]. *POLE* mutated tumors constitute about 10% of our EC cohort and are associated with excellent outcome. They are characterized by high grade, low stage, specific myometrial invasion patterns (MELF and tumor budding), intense intra- and peri-tumoral lymphocytic infiltrate, and high proliferative activity. We confirm the link reported in other studies between *POLE* tumors and lower BMI, although in our series there is no statistical association to young age at the time of diagnosis [43].

The MMRd group (approximately 30% of our cases) shows morphological characteristics similar to those of the *POLE* group, such as endometrioid-type histology and abundance of tumor-infiltrating lymphocytes. However, MMRd ECs have an intermediate prognosis, which significantly differs from that of the *POLE* group tumors.

Tumors of the p53 abnormal group (approximately 20% in our series) have aggressive histologic characteristics including high grade, non-endometrioid features, and significantly higher FIGO stage. NSMP ECs represent approximately 40% of the cases in our cohort and are predominantly low-grade endometrioid carcinomas with low proliferative activity. Although our results are consistent with literature, the classification into four molecular TCGA groups alone did not achieve statistical significance in prognostic stratification.

Our study was not limited only to surrogate TCGA subtyping, but it also aimed to investigate the relevance of *ARID1A* and *CTNNB1* mutations and their predictive-prognostic impact with particular reference to the NSMP group.

*CTNNB1* mutations/β-catenin abnormal expression (found in approximately 20% of NSMP tumors) identify a subset characterized by young age at diagnosis, high BMI, low mitotic rate, low Ki67 proliferative index, and low TILs counts. As reported in the literature, *CTNNB1* mutated tumors are predominantly low grade and low stage, but in our cohort this molecular alteration is not linked to unfavorable prognosis.

A relevant finding of our study is the definition of the clinical and prognostic impact of ARID1A alterations. ARID1A normally maintains endometrial epithelial cell identity by repressing mesenchymal cell fates. A recent study has shown that coexistent *ARID1A* and *PI3K* mutations promote epithelial transdifferentiation associated with epithelial-to-mesenchymal transition (EMT) [19]. These findings support a tumor suppressor role for ARID1A-containing SWI/SNF complexes, so its loss-of-function may increase the EC invasive potential. Previous studies have also shown that ARID1A alteration is strongly associated with sporadic mismatch repair loss [16,17], suggesting that by having a role in epigenetic silencing of *MLH1, ARID1A* is a causative, instead of a target gene for microsatellite instability. However, in our MMRd group, ARID1A loss is usually a subclonal event—both by IHC and NGS—suggesting that the alteration of ARID1A follows, instead of preceding microsatellite instability. In our study we have confirmed that ARID1A alterations occur in both MMRd as well as *POLE* group tumors, while they are inversely related to p53 mutated tumors. In the entire cohort, *ARID1A* mutated carcinomas are prevalently endometrioid, undifferentiated/dedifferentiated and exhibit histopathologic features such as MELF, presence of TILs and high proliferative index. We have explored the role of ARID1A in the β-catenin altered and NSMP subgroups to determine its impact on clinical features and prognosis. In both subgroups ARID1A alteration is associated with novel and distinctive histological features: (1) in β-catenin altered tumors, ARID1A loss correlates with high histologic grade, necrosis, tumor budding, TILs and high proliferative activity; (2) in the NSMP group, *ARID1A* mutation correlates with increased proliferative activity and, interestingly, it identified all NSMP with recurrent disease. This remarkable finding can improve the surrogate molecular EC classification differentiating the biological heterogeneity of NSMP tumors and identifying a subset of ECs (NSMP_A) at higher risk of relapse. By integrating conventional ESMO 2016 clinicopathologic criteria and narrowing the analysis to high risk groups, our immuno-molecular classifier implemented with β-catenin and ARID1A alterations proved to be statistically associated with recurrence. In order to test the performance of our immuno-molecular classification with an external case series we tried our algorithm using TCGA data (see Figure S2). The log-rank test showed a trend for both overall and disease-free survival (*p*-value of 0.069 and of 0.081, respectively), indicating conformity with TCGA data. However, as also shown in previous studies, surrogate molecular classification is similar to—but does not simply overlap with—the TCGA scheme for endometrial cancer [5,6,7]. In particular, we—unlike TCGA—have selected high-risk cases according to ESMO criteria based on clinicopathologic features and then tested material from these cases for *ARID1A* and *CTNNB1*/β-catenin. In addition, our study has included immunohistochemical analysis for ARID1A on whole slides in order to identify subclones and to guide subsequent molecular sequencing. Moreover, our cases have been enrolled consecutively, without selection bias, from a referral center for gynecologic oncology, with survival time and follow-up different from the TCGA cases. These points may explain some of the differences in survival patterns when our classifier is applied to TCGA data (see Figure S2).

The multistep classification approach proposed in our study allows to better discriminate NSMP tumors by the simple addition of two markers to the already known PORTEC/ProMisE algorithms. In particular, the assessment of ARID1A in NSMP group could change the clinical management of these patients: i.e., a closer follow-up could be proposed for an early detection of possible recurrence. In addition, ARID1A is emerging as a potential therapeutic target. Recent studies have showed that ARID1A loss is associated with improved response to immunotherapy across diverse tumor types [44,45]. Considering these observations, the presence of ARID1A alterations may enable better patient selection who benefit from immune checkpoint blockade, also in non-*POLE*/MMRd tumors.

## 5. Conclusions

The evolution of EC classification from being purely based on morphology, to classification incorporating molecular profile promises for more accurately subtyping endometrial carcinoma to better reflect patient prognosis and outcome. This study confirms the feasibility of surrogate molecular TCGA classification of EC into routine clinical practice. Our immuno-molecular classification scheme supplemented with NSMP tumor sub-grouping based on the *ARID1A* and *CTNNB1* status granted a more reliable risk assessment and it resulted to be particularly informative in the group of high-risk patients. Our data indicates that *ARID1A* analysis may be a useful biomarker to identify patients who have worse prognosis in the NSMP group and may therefore require more aggressive forms of treatment and closer follow-up. 

However, this classifier does not replace risk assessment based on conventional clinicopathologic parameters that will remain essential in prognostic stratification (i.e., FIGO stage). It is reasonable that molecular and clinicopathologic prognostic grouping systems will likely work better together.

## Figures and Tables

**Table 1:** Clinicopathologic characteristics of the study sample. Values are counts (percentages) or mean ± standard deviation [interquartile range].

Clinicopathologic Characteristics	*n* = 125 (%)
Age, years	62.7 ± 10.7
	[56–71]
Body mass index, kg/m^2^	27.5 ± 6.6
	[22.8–30.1]
Tumor type	
Endometrioid	90 (72.0)
Dedifferentiated/Undifferentiated	17 (13.6)
Serous	15 (12.0)
Clear cell	3 (2.4)
Grade	
1	36 (28.8)
2	35 (28.0)
3	54 (43.2)
Depth of invasion	
<50%	90 (72.0)
≥50%	35 (28.0)
Lymphovascular space invasion (LVSI)	
Absent	88 (70.4)
Present	37 (29.6)
Lymph node status	
Negative	95 (76.0)
Positive	24 (19.2)
Unknown/Not tested	6 (4.8)
FIGO stage	
IA	71 (56.8)
IB	18 (14.4)
II	4 (3.2)
III	30 (24.0)
IV	2 (1.6)
ESMO (2016)	
Low	18 (14.4)
Intermediate	8 (6.4)
High–Intermediate	42 (33.6)
High	57 (45.6)
Extensive necrosis	
Absent	66 (52.8)
Present	59 (47.2)
MELF	
Absent	79 (63.2)
Present	46 (36.8)
Tumor budding	
Absent	73 (58.4)
Present	52 (41.6)
sTILs	
Low	36 (28.8)
High	89 (71.2)
iTILs	
Low	39 (31.2)
High	86 (68.8)
Recurrence	
Absent	114 (91.2)
Present	11 (8.8)

**Table 2:** Clinicopathologic characteristics of surrogate TGCA molecular groups. Values are counts (percentages) or mean ± standard deviation [interquartile range].

Clinicopathologic Characteristics	*POLE*	MMRd	p53abn	NSMP	*p*-Value
	(n = 9; 7.2%)	(n = 41; 32.8%)	(n = 26; 20.8%)	(n = 49; 39.2%)	
Age, years	61.2 ± 13.9	64.2 ± 10.0	65.0 ± 10.0	60.6 ± 11.0	0.266
	[52–71]	[57–73]	[59–74]	[55–69]	
Body mass index, kg/m^2^	25.3 ± 4.6	26.8 ± 6.1	25.4 ± 4.1	29.5 ± 7.8	0.104
	[21.3–28.1]	[22.7–28.2]	[22.8–27.2]	[24.0–33.8]	
Tumor type					<0.001
Endometrioid	8 (88.9)	30 (73.2)	7 (26.9)	45 (91.8)	
Dedifferentiated/ Undifferentiated	1 (11.1)	11 (26.8)	1 (3.8)	4 (8.2)	
Serous	0 (0.0)	0 (0.0)	15 (57.7)	0 (0.0)	
Clear cell	0 (0.0)	0 (0.0)	3 (11.5)	0 (0.0)	
Heterogeneity	4 (44.4)	21 (51.2)	10 (38.5)	14 (28.6)	0.168
Grade					<0.001
1	2 (22.2)	11 (26.8)	1 (3.8)	22 (44.9)	
2	3 (33.3)	14 (34.1)	0 (0.0)	18 (36.7)	
3	4 (44.4)	16 (39.0)	25 (96.2)	9 (18.4)	
Depth of invasion ≥50%	1 (11.1)	15 (36.6)	9 (34.6)	10 (20.4)	0.208
LVSI	2 (22.2)	14 (34.1)	11 (42.3)	10 (20.4)	0.196
Lymph node status					0.141
Negative	8 (88.9)	31 (75.6)	15 (57.7)	41 (83.7)	
Positive	1 (11.1)	8 (19.5)	10 (38.5)	5 (10.2)	
FIGO stage					0.011
IA	5 (55.6)	19 (46.3)	12 (46.2)	35 (71.4)	
IB/II	2 (22.2)	12 (29.3)	1 (3.8)	7 (14.3)	
III	2 (22.2)	9 (22.0)	12 (46.2)	7 (14.3)	
IV	0 (0.0)	1 (2.4)	1 (3.8)	0 (0.0)	
Extensive necrosis	6 (66.7)	23 (56.1)	11 (42.3)	19 (38.8)	0.235
MELF	5 (55.6)	24 (58.5)	3 (11.5)	14 (28.6)	<0.001
Tumor budding	7 (77.8)	23 (56.1)	8 (30.8)	14 (28.6)	0.004
High sTILs	9 (100.0)	35 (85.4)	19 (73.1)	26 (53.1)	0.001
High iTILs	9 (100.0)	37 (90.2)	17 (65.4)	23 (46.9)	<0.001
Mitoses/10 HPF	78.1 ± 34.5	55.3 ± 23.7	86.3 ± 43.7	33.3 ± 27.7	<0.001
	[50–103]	[40–70]	[45–130]	[10–42]	
Ki67 proliferative index	58.5 ± 14.9	57.9 ± 14.9	56.0 ± 16.7	36.6 ± 18.3	<0.001
	[55.1–68.2]	[47.1–69.6]	[49.3–69.7]	[23.3–50.0]	

**Table 3:** Surrogate TCGA molecular groups including β-catenin altered subgroup. Values are counts (percentages) or mean ± standard deviation [interquartile range].

Clinicopathologic Characteristics	*POLE*	MMRd	p53abn	β-CATabn	NSMP	*p*-Value
	(*n* = 9; 7.2%)	(*n* = 41; 32.8%)	(*n* = 26; 20.8%)	(*n* = 16; 12.8%)	(*n* = 33; 26.4%)	
Age, years	61.2 ± 13.9	64.2 ± 10.0	65.0 ± 10.0	54.3 ± 12.9	63.6 ± 8.5	0.067
	[52–71]	[57–73]	[59–74]	[44–63]	[58–70]	
Body mass index, kg/m^2^	25.3 ± 4.6	26.8 ± 6.1	25.4 ± 4.1	29.0 ± 6.7	29.7 ± 8.3	0.187
	[21.3–28.1]	[22.7–28.2]	[22.8–27.2]	[22.5–33.5]	[24.2–36.1]	
Tumor type						<0.001
Endometrioid	8 (88.9)	30 (73.2)	7 (26.9)	14 (87.5)	31 (93.9)	
Dedifferentiated/ Undifferentiated	1 (11.1)	11 (26.8)	1 (3.8)	2 (12.5)	2 (6.1)	
Serous	0 (0.0)	0 (0.0)	15 (57.7)	0 (0)	0 (0)	
Clear cell	0 (0.0)	0 (0.0)	3 (11.5)	0 (0)	0 (0)	
Heterogeneity	4 (44.4)	21 (51.2)	10 (38.5)	5 (31.3)	9 (27.3)	0.290
Grade						<0.001
1	2 (22.2)	11 (26.8)	1 (3.8)	8 (50.0)	14 (42.4)	
2	3 (33.3)	14 (34.1)	0 (0.0)	5 (31.3)	13 (39.4)	
3	4 (44.4)	16 (39.0)	25 (96.2)	3 (18.8)	6 (18.2)	
Depth of invasion ≥50%	1 (11.1)	15 (36.6)	9 (34.6)	3 (18.8)	7 (21.2)	0.361
LVSI	2 (22.2)	14 (34.1)	11 (42.3)	3 (18.8)	7 (21.2)	0.350
Lymph node status						0.193
Negative	8 (88.9)	31 (75.6)	15 (57.7)	15 (93.8)	26 (78.8)	
Positive	1 (11.1)	8 (19.5)	10 (38.5)	1 (6.3)	4 (12.1)	
Unknown/Not tested	0 (0.0)	2 (4.9)	1 (3.8)	0 (0.0)	3 (9.1)	
FIGO stage						0.048
IA	5 (55.6)	19 (46.3)	12 (46.2)	11 (68.8)	24 (72.7)	
IB/II	2 (22.2)	12 (29.3)	1 (3.8)	3 (18.8)	4 (12.1)	
III	2 (22.2)	9 (22.0)	12 (46.2)	2 (12.5)	5 (15.2)	
IV	0 (0.0)	1 (2.4)	1 (3.8)	0 (0.0)	0 (0.0)	
Extensive necrosis	6 (66.7)	23 (56.1)	11 (42.3)	4 (25.0)	15 (45.5)	0.195
MELF	5 (55.6)	24 (58.5)	3 (11.5)	4 (25.0)	10 (30.3)	0.001
Tumor budding	7 (77.8)	23 (56.1)	8 (30.8)	4 (25.0)	10 (30.3)	0.011
High sTILs	9 (100.0)	35 (85.4)	19 (73.1)	7 (43.8)	19 (57.6)	0.002
High iTILs	9 (100.0)	37 (90.2)	17 (65.4)	4 (25.0)	19 (57.6)	<0.001
Mitoses/10 HPF	78.1 ± 34.5	55.3 ± 23.7	86.3 ± 43.7	30.5 ± 30.1	34.6 ± 26.8	<0.001
	[50–103]	[40–70]	[45–130]	[9–44]	[20–40]	
Ki67 proliferative index	58.5 ± 14.9	57.9 ± 14.9	56.0 ± 16.7	35.9 ± 21.6	36.9 ± 16.6	<0.001
	[55.1–68.2]	[47.1–69.6]	[49.3–69.7]	[17.1–58.4]	[25.3–42.7]	

**Table 4:** NSMP subgroups by β-catenin and *ARID1A* alterations. Values are counts (percentages) or mean ± standard deviation [interquartile range].

Clinicopathologic Characteristics	β-Catenin Abnormal Subgroup (*n* = 16; 32,7%)			NSMP Subgroup (*n* = 33; 67,3%)		
	β-CATabn	β-CATabn_A	*p*-Value	NSMP	NSMP_A	*p*-Value
	(*n* = 9)	(*n* = 7)		(*n* = 14)	(*n* = 19)	
Age, years	49 ± 13	61 ± 10	0.044	66 ± 10	62 ± 7	0.352
	[40–54]	[55–73]		[55–75]	[58–69]	
Body mass index, kg/m^2^	27.3 ± 7.8	31.1 ± 4.7	0.186	27.6 ± 6.1	31.3 ± 9.5	0.229
	[20.3–29.1]	[28.1–33.8]		[23.4–28.9]	[24.2–37.3]	
Tumor type			0.175			1.000
Endometrioid	9 (100)	5 (71)		13 (93)	18 (95)	
Dedifferentiated/Undifferentiated	0 (0)	2 (29)		1 (7)	1 (5)	
Heterogeneity	1 (11)	4 (57)	0.106	2 (14)	7 (37)	0.241
Grade			0.001			0.391
1	8 (89)	0 (0)		8 (57)	6 (32)	
2	1 (11)	4 (57)		4 (29)	9 (47)	
3	0 (0)	3 (43)		2 (14)	4 (21)	
Depth of invasion ≥50%	0 (0)	3 (43)	0.062	3 (21)	4 (21)	1.000
LVSI	0 (0)	3 (43)	0.062	2 (14)	5 (26)	0.670
Lymph node status			0.437			0.830
Negative	9 (100)	6 (86)		12 (86)	14 (74)	
Positive	0 (0)	1 (14)		1 (7)	3 (16)	
Unknown/Not tested	0 (0)	0 (0)		1 (7)	2 (11)	
FIGO stage			0.758			1.000
IA	7 (78)	4 (57)		10 (71)	14 (74)	
IB/II	1 (11)	2 (29)		2 (14)	2 (11)	
III	1 (11)	1 (14)		2 (14)	3 (16)	
Extensive necrosis	0 (0)	4 (57)	0.019	6 (43)	9 (47)	1.000
MELF	2 (22)	2 (29)	1.000	3 (21)	7 (37)	0.455
Tumor budding	0 (0)	4 (57)	0.019	3 (21)	7 (37)	0.455
High sTILs	1 (11)	6 (86)	0.009	7 (50)	12 (63)	0.497
High iTILs	0 (0)	4 (57)	0.019	6 (43)	13 (68)	0.173
Mitoses/10 HPF	9.3 ± 4.8	57.7 ± 26.3	0.001	31.1 ± 33.7	37.2 ± 21.0	0.100
	[5–10]	[42–80]		[10–40]	[24–50]	
Ki67 proliferative index	20.7 ± 11.2	55.4 ± 14.5	0.003	31.4 ± 18.8	41.5 ± 13.5	0.037
	[13.8–29.6]	[49.5–64.8]		[19.3–36.9]	[33.7–42.7]	

## Data Availability

MDPI Research Data Policies.

## Supplementary Materials

The following are available online at https://www.mdpi.com/2072-6694/13/5/950/s1, Figure S1: Kaplan–Meier curves in patients at high-intermediate and high-risk according to ESMO by surrogate TCGA molecular groups; Table S1: List of antibodies; Table S2: Clinicopathologic characteristics of *CTNNB1* mutated/β-catenin abnormal versus *CTNNB1* wild-type/β-catenin normal carcinomas; Table S3: Clinicopathologic characteristics of *ARID1A* altered versus *ARID1A* wild-type carcinomas; Figure S2: Kaplan–Meier estimates of disease-free survival and overall survival in TCGA case series, by molecular subgroup including β-catenin and ARID1A alterations.

## References

[1] Ferlay J., Colombet M., Soerjomataram I., Dyba T., Randi G., Bettio M., Gavin A., Visser O., Bray F. (2018). Cancer incidence and mortality patterns in Europe: Estimates for 40 countries and 25 major cancers in 2018. Eur. J. Cancer.

[2] Allemani C., Matsuda T., Di Carlo V., Harewood R., Matz M., Nikšić M., Bonaventure A., Valkov M., Johnson C.J., Estève J. (2018). Global surveillance of trends in cancer survival 2000-14 (CONCORD-3): Analysis of individual records for 37 513 025 patients diagnosed with one of 18 cancers from 322 population-based registries in 71 countries. Lancet.

[3] Gilks C.B., Oliva E., Soslow R.A. (2013). Poor interobserver reproducibility in the diagnosis of high-grade endometrial carcinoma. Am. J. Surg. Pathol..

[4] Kandoth C., Schultz N., Cherniack A.D., Akbani R., Liu Y., Shen H., Robertson A.G., Pashtan I., Shen R., Benz C.C. (2013). Integrated genomic characterization of endometrial carcinoma. Nature.

[5] Stelloo E., Nout R.A., Osse E.M., Jurgenliemk-Schulz I.J., Jobsen J.J., Lutgens L.C., van der Steen-Banasik E.M., Nijman H.W., Putter H., Bosse T. (2016). Improved Risk Assessment by Integrating Molecular and Clinicopathological Factors in Early-stage Endometrial Cancer-Combined Analysis of the PORTEC Cohorts. Clin. Cancer Res..

[6] Talhouk A., McConechy M.K., Leung S., Li-Chang H.H., Kwon J.S., Melnyk N., Yang W., Senz J., Boyd N., Karnezis A.N. (2015). A clinically applicable molecular-based classification for endometrial cancers. Br. J. Cancer.

[7] Talhouk A., McConechy M.K., Leung S., Yang W., Lum A., Senz J., Boyd N., Pike J., Anglesio M., Kwon J.S. (2017). Confirmation of ProMisE: A simple, genomics-based clinical classifier for endometrial cancer. Cancer.

[8] Kurnit K.C., Kim G.N., Fellman B.M., Urbauer D.L., Mills G.B., Zhang W., Broaddus R.R. (2017). CTNNB1 (beta-catenin) mutation identifies low grade, early stage endometrial cancer patients at increased risk of recurrence. Mod. Pathol..

[9] Liu Y., Patel L., Mills G.B., Lu K.H., Sood A.K., Ding L., Kucherlapati R., Mardis E.R., Levine D.A., Shmulevich I. (2014). Clinical significance of CTNNB1 mutation and Wnt pathway activation in endometrioid endometrial carcinoma. J. Natl. Cancer Inst..

[10] Wilson B.G., Roberts C.W. (2011). SWI/SNF nucleosome remodellers and cancer. Nat. Rev. Cancer..

[11] Shen J., Peng Y., Wei L., Zhang W., Yang L., Lan L., Kapoor P., Ju Z., Mo Q., Shih I. (2015). ARID1A Deficiency Impairs the DNA Damage Checkpoint and Sensitizes Cells to PARP Inhibitors. Cancer Discov..

[12] Mathur R. (2018). ARID1A loss in cancer: Towards a mechanistic understanding. Pharmacol Ther..

[13] Guan B., Mao T.L., Panuganti P.K., Kuhn E., Kurman R.J., Maeda D., Chen E., Jeng Y.M., Wang T.L., Shih I. (2011). Mutation and loss of expression of ARID1A in uterine low-grade endometrioid carcinoma. Am. J. Surg. Pathol..

[14] Rahman M., Nakayama K., Rahman M.T., Katagiri H., Katagiri A., Ishibashi T., Ishikawa M., Iida K., & Miyazaki K. (2013). Clinicopathologic analysis of loss of AT-rich interactive domain 1A expression in endometrial cancer. Hum. Pathol..

[15] Wiegand K.C., Lee A.F., Al-Agha O.M., Chow C., Kalloger S.E., Scott D.W., Steidl C., Wiseman S.M., Gascoyne R.D., Gilks B. (2011). Loss of BAF250a (ARID1A) is frequent in high-grade endometrial carcinomas. J. Pathol..

[16] Allo G., Bernardini M.Q., Wu R.C., Shih I.M., Kalloger S., Pollett A., Gilks C.B., Clarke B.A. (2014). ARID1A loss correlates with mismatch repair deficiency and intact p53 expression in high-grade endometrial carcinomas. Mod. Pathol..

[17] Bosse T., ter Haar N.T., Seeber L.M., Diest P.J.V., Hes F.J., Vasen H.F., Nout R.A., Creutzberg C.L., Morreau H., Smit V.T. (2013). Loss of ARID1A expression and its relationship with PI3K-Akt pathway alterations, TP53 and microsatellite instability in endometrial cancer. Mod. Pathol..

[18] Yen T.T., Miyamoto T., Asaka S., Chui M.H., Wang Y., Lin S.F., Stone R.L., Fader A.N., Asaka R., Kashima H. (2018). Loss of ARID1A expression in endometrial samplings is associated with the risk of endometrial carcinoma. Gynecol. Oncol..

[19] Wilson M.R., Reske J.J., Holladay J., Wilber G.E., Rhodes M., Koeman J., Adams M., Johnson B., Su R.W., Joshi N.R. (2019). ARID1A and PI3-kinase pathway mutations in the endometrium drive epithelial transdifferentiation and collective invasion. Nat. Commun..

[20] Colombo N., Creutzberg C., Amant F., Bosse T., Gonzalez-Martin A., Ledermann J., Marth C., Nout R., Querleu D., Mirza M.R. (2016). ESMO-ESGO-ESTRO Consensus Conference on Endometrial Cancer: Diagnosis, treatment and follow-up. Ann. Oncol..

[21] Perrone A.M., Di Marcoberardino B., Rossi M., Pozzati F., Pellegrini A., Procaccini M., Santini D., De Iaco P. (2012). Laparoscopic versus laparotomic approach to endometrial cancer. Eur. J. Gynaecol. Oncol..

[22] The WHO Classification of Tumours Editorial Board (2020). WHO Classification of Tumours, Female Genital Tumours.

[23] (2006). FIGO: 27th volume of the ANNUAL REPORT on the Results of Treatment in Gynecological Cancer. Int. J. Gynaecol. Obstet..

[24] Murray S.K., Young R.H., Scully R.E. (2003). Unusual epithelial and stromal changes in myoinvasive endometrioid adenocarcinoma: A study of their frequency, associated diagnostic problems, and prognostic significance. Int. J. Gynecol. Pathol..

[25] Euscher E., Fox P., Bassett R., Al-Ghawi H., Ali-Fehmi R., Barbuto D., Djordjevic B., Frauenhoffer E., Kim I., Hong S.R. (2013). The pattern of myometrial invasion as a predictor of lymph node metastasis or extrauterine disease in low-grade endometrial carcinoma. Am. J. Surg. Pathol..

[26] Bosse T., Peters E.E., Creutzberg C.L., Jurgenliemk-Schulz I.M., Jobsen J.J., Mens J.W., Lutgens L.C., van der Steen-Banasik E.M., Smit V.T., Nout R.A. (2015). Substantial lymph-vascular space invasion (LVSI) is a significant risk factor for recurrence in endometrial cancer--A pooled analysis of PORTEC 1 and 2 trials. Eur. J. Cancer.

[27] Fujimoto T., Nanjyo H., Fukuda J., Nakamura A., Mizunuma H., Yaegashi N., Sugiyama T., Kurachi H., Sato A., Tanaka T. (2009). Endometrioid uterine cancer: Histopathological risk factors of local and distant recurrence. Gynecol. Oncol..

[28] Shia J., Black D., Hummer A.J., Boyd J., Soslow R.A. (2008). Routinely assessed morphological features correlate with microsatellite instability status in endometrial cancer. Hum. Pathol..

[29] Hendry S., Salgado R., Gevaert T., Russell P.A., John T., Thapa B., Christie M., van de Vijver K., Estrada M.V., Gonzalez-Ericsson P.I. (2017). Assessing Tumor-Infiltrating Lymphocytes in Solid Tumors: A Practical Review for Pathologists and Proposal for a Standardized Method from the International Immuno-Oncology Biomarkers Working Group: Part *2*: TILs in Melanoma, Gastrointestinal Tract Carcinomas, Non-Small Cell Lung Carcinoma and Mesothelioma, Endometrial and Ovarian Carcinomas, Squamous Cell Carcinoma of the Head and Neck, Genitourinary Carcinomas, and Primary Brain Tumors. Adv. Anat. Pathol..

[30] Singh N., Piskorz A.M., Bosse T., Jimenez-Linan M., Rous B., Brenton J.D., Gilks C.B., Köbel M. (2020). p53 immunohistochemistry is an accurate surrogate for TP53 mutational analysis in endometrial carcinoma biopsies. J. Pathol..

[31] Garg K., Broaddus R.R., Soslow R.A., Urbauer D.L., Levine D.A., Djordjevic B. (2012). Pathologic scoring of PTEN immunohistochemistry in endometrial carcinoma is highly reproducible. Int. J. Gynecol. Pathol..

[32] Nei H., Saito T., Yamasaki H., Mizumoto H., Ito E., Kudo R. (1999). Nuclear localization of beta-catenin in normal and carcinogenic endometrium. Mol. Carcinog..

[33] Travaglino A., Raffone A., Saccone G., De Luca C., Mollo A., Mascolo M., De Placido G., Insabato L., Zullo F. (2019). Immunohistochemical Nuclear Expression of beta-Catenin as a Surrogate of CTNNB1 Exon 3 Mutation in Endometrial Cancer. Am. J. Clin. Pathol..

[34] Dondi G., Coluccelli S., De Leo A., Ferrari S., Gruppioni E., Bovicelli A., Godino L., Coadă C.A., Morganti A.G., Giordano A. (2020). An Analysis of Clinical, Surgical, Pathological and Molecular Characteristics of Endometrial Cancer According to Mismatch Repair Status. A Multidisciplinary Approach. Int. J. Mol. Sci..

[35] de Biase D., Acquaviva G., Visani M., Sanza V., Argento C.M., De Leo A., Maloberti T., Pession A., Tallini G. (2020). Molecular Diagnostic of Solid Tumor Using a Next Generation Sequencing Custom-Designed Multi-Gene Panel. Diagnostics.

[36] van Roon E.H., van Puijenbroek M., Middeldorp A., van Eijk R., de Meijer E.J., Erasmus D., Wouters K.A., van Engeland M., Oosting J., Hes F.J. (2010). Early onset MSI-H colon cancer with MLH1 promoter methylation, is there a genetic predisposition?. BMC Cancer.

[37] Leon-Castillo A., Britton H., McConechy M.K., McAlpine J.N., Nout R., Kommoss S., Brucker S.Y., Carlson J.W., Epstein E., Rau T.T. (2020). Interpretation of somatic POLE mutations in endometrial carcinoma. J. Pathol..

[38] Kommoss S., McConechy M.K., Kommoss F., Leung S., Bunz A., Magrill J., Britton H., Kommoss F., Grevenkamp F., Karnezis A. (2018). Final validation of the ProMisE molecular classifier for endometrial carcinoma in a large population-based case series. Ann. Oncol..

[39] Stelloo E., Bosse T., Nout R.A., MacKay H.J., Church D.N., Nijman H.W., Leary A., Edmondson R.J., Powell M.E., Crosbie E.J. (2015). Refining prognosis and identifying targetable pathways for high-risk endometrial cancer; a TransPORTEC initiative. Mod. Pathol..

[40] Raffone A., Travaglino A., Mascolo M., Carbone L., Guida M., Insabato L., Zullo F. (2019). TCGA molecular groups of endometrial cancer: Pooled data about prognosis. Gynecol. Oncol..

[41] Espinosa I., De Leo A., D’Angelo E., Rosa-Rosa J.M., Corominas M., Gonzalez A., Palacios J., Prat J. (2018). Dedifferentiated endometrial carcinomas with neuroendocrine features: A clinicopathologic, immunohistochemical, and molecular genetic study. Hum. Pathol..

[42] Kim G., Kurnit K.C., Djordjevic B., Singh C., Munsell M.F., Wang W.L., Lazar A.J., Zhang W., Broaddus R. (2018). Nuclear beta-catenin localization and mutation of the CTNNB1 gene: A context-dependent association. Mod. Pathol..

[43] Roque D.R., Makowski L., Chen T.H., Rashid N., Hayes D.N., Bae-Jump V. (2016). Association between differential gene expression and body mass index among endometrial cancers from The Cancer Genome Atlas Project. Gynecol. Oncol..

[44] Shen J., Ju Z., Zhao W., Wang L., Peng Y., Ge Z., Nagel Z.D., Zou J., Wang C., Kapoor P. (2018). ARID1A deficiency promotes mutability and potentiates therapeutic antitumor immunity unleashed by immune checkpoint blockade. Nat. Med..

[45] Okamura R., Kato S., Lee S., Jimenez R.E., Sicklick J.K., Kurzrock R. (2020). ARID1A alterations function as a biomarker for longer progression-free survival after anti-PD-1/PD-L1 immunotherapy. J. Immunother Cancer..

