# Supplementary material for: ARID1A and CTNNB1/β-Catenin Molecular Status Affects the Clinicopathologic Features and Prognosis of Endometrial Carcinoma: Implications for an Improved Surrogate Molecular Classification
Source: Cancers (Basel). 2021 Feb 25;13(5):950. doi: 10.3390/cancers13050950 (PMC7956405; doi:10.3390/cancers13050950)
Supplement: Supplementary file 1 [file cancers-13-00950-s001.pdf]

Supplementary materials

Table S1: List of antibodies

Antibody	Source	Dilution	Method
Mouse anti-β-Catenin, clone 14	Cell Marque, USA	RTU	UltraCC1 x 32' at 95 ℃ - Ab 16' at 36 ℃
Rabbit anti-PTEN, clone SP218	Ventana, USA	RTU	UltraCC1 x 56' at 100 ℃ - Ab 16' at 36 ℃
Rabbit anti-ARID1A polyclonal	Atlas antibodies	1:90	UltraCC1 x 32' at 95 ℃ - Ab 32' at 36 ℃
Mouse anti-p53, clone DO7	Ventana, USA	RTU	UltraCC1 x 24' at 95 ℃ - Ab 12' at 36 ℃
Mouse anti-MLH1, clone M1	Ventana, USA	RTU	UltraCC1 x 56' at 98 ℃ - Ab 32' at 36 ℃
Mouse anti-PMS2, clone A16-4	Ventana, USA	RTU	UltraCC1 x 64' at 99 ℃ - Ab 32' at 36 ℃
Mouse anti-MSH2, clone G219-1129	Ventana, USA	RTU	UltraCC1 x 56' at 95 ℃ - Ab 32' at 36 ℃
Rabbit anti-MSH6, clone SP93	Ventana, USA	RTU	UltraCC1 x 64' at 100 ℃ - Ab 12' at 36 ℃
Rabbit anti-Ki67, clone 30-9	Ventana, USA	RTU	UltraCC1 x 32' at 99 ℃ - Ab 8' at RT

Visualization with OptiView DAB Detection kit, Ventana, USA

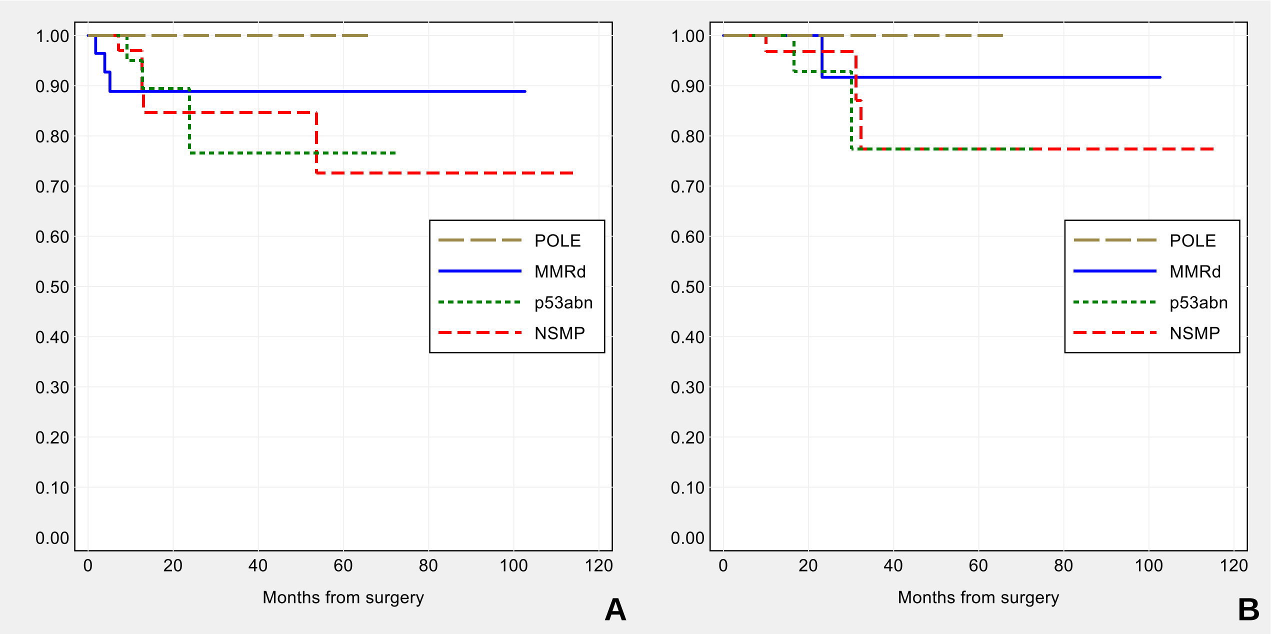

Figure S1. Kaplan–Meier estimates of disease-free survival (A) and overall survival (B) in patients at high-intermediate and high-risk according to ESMO (n = 99), by surrogate TCGA molecular groups.

Table S2. Clinicopathologic characteristics of *CTNNB1* mutated/β-catenin abnormal versus *CTNNB1* wild-type/β-catenin normal carcinomas. Values are counts (percentages) or mean ± standard deviation [interquartile range].

**Characteristics**	***CTNNB1* mutation/ β-catenin abnormal** (*n* = 21; 16.8%)	***CTNNB1* wild-type/ β-catenin normal** (*n* = 104; 83.2%)	***P*-value**
Age, years	54.9 ± 12.9 [44–64]	64.3 ± 9.6 [58–71.5]	<0.001
Body mass index, kg/m ^{2}	30.6 ± 7.8 [24.6–34.2]	26.8 ± 6.1 [22.7–29.3]	0.016
Tumor type			0.224
Endometrioid	17 (81.0)	73 (70.2)	
Dedifferentiated/Undifferentiated	4 (19.0)	13 (12.5)	
Serous	0 (0.0)	15 (14.4)	
Clear cell	0 (0.0)	3 (2.9)	
TGCA molecular group			0.001
POLE	1 (4.8)	8 (7.7)	
MMRd	4 (19.0)	37 (35.6)	
p53abn	0 (0.0)	26 (25.0)	
NSMP	16 (76.2)	33 (31.7)	
Heterogeneity	7 (33.3)	42 (40.4)	0.546
Grade			0.071
1	10 (47.6)	26 (25.0)	
2	6 (28.6)	29 (27.9)	
3	5 (23.8)	49 (47.1)	
Depth of invasion ≥50%	5 (23.8)	30 (28.8)	0.639
Lymphovascular space invasion	5 (23.8)	32 (30.8)	0.524
Lymph node status			0.260
Negative	19 (90.5)	76 (73.1)	
Positive	2 (9.5)	22 (21.2)	
Unknown/Not tested	0 (0.0)	6 (5.8)	
FIGO stage			0.663
IA	14 (66.7)	57 (54.8)	
IB/II	4 (19.0)	18 (17.3)	
III	3 (14.3)	27 (26.0)	
IV	0 (0.0)	2 (1.9)	
Extensive necrosis	7 (33.3)	52 (50.0)	0.163
MELF	7 (33.3)	39 (37.5)	0.718
Tumor budding	6 (28.6)	46 (44.2)	0.184
High sTILs	11 (52.4)	78 (75.0)	0.037
High iTILs	9 (42.9)	77 (74.0)	0.005
Mitoses/10 HPF	37.6 ± 32.0 [10–60]	58.2 ± 37.1 [30–81]	0.019
Ki67 proliferation index	41.8 ± 22.5 [19.7–63.1]	50.9 ± 18.3 [38.0–67.6]	0.049

Table S3. Clinicopathologic characteristics of *ARID1A* altered versus *ARID1A* wild-type carcinomas. Values are counts (percentages) or mean ± standard deviation [interquartile range].

**Clinicopathologic Characteristics**	***ARID1A* altered** (*n* = 69; 55.2%)	***ARID1A* wild-type** (*n* = 56; 44.8%)	***P*-value**
Age, years	63.0 ± 9.6 [56–71]	62.4 ± 12.0 [55–72]	0.742
Body mass index, kg/m ^{2}	27.9 ± 6.8 [22.8–31.1]	26.9 ± 6.3 [22.6–29.1]	0.362
Tumor type			0.002
Endometrioid	54 (78.3)	36 (64.3)	
Dedifferentiated/ Undifferentiated	12 (17.4)	5 (8.9)	
Serous	3 (4.3)	12 (21.4)	
Clear cell	0 (0.0)	3 (5.4)	
TGCA molecular group			<0.001
POLE	8 (11.6)	1 (1.8)	
MMRd	33 (47.8)	8 (14.3)	
p53abn	3 (4.3)	23 (41.1)	
NSMP	25 (36.2)	24 (42.9)	
Heterogeneity	32 (46.4)	17 (30.4)	0.068
Grade			0.028
1	17 (24.6)	19 (33.9)	
2	26 (37.7)	9 (16.1)	
3	26 (37.7)	28 (50.0)	
Depth of invasion ≥50%	22 (31.9)	13 (23.2)	0.283
LVSI	25 (36.2)	12 (21.4)	0.071
Lymph node status			0.344
Negative	50 (72.5)	45 (80.4)	
Positive	14 (20.3)	10 (17.9)	
FIGO stage			0.850
IA	38 (55.1)	33 (58.9)	
IB/II	14 (20.3)	8 (14.3)	
III	16 (23.2)	14 (25.0)	
IV	1 (1.4)	1 (1.8)	
Extensive necrosis	35 (50.7)	24 (42.9)	0.381
MELF	36 (52.2)	10 (17.9)	<0.001
Tumor budding	35 (50.7)	17 (30.4)	0.022
High sTILs	56 (81.2)	33 (58.9)	0.006
High iTILs	57 (82.6)	29 (51.8)	<0.001
Mitoses/10 HPF	56.0 ± 29.6 [32–70]	53.2 ± 44.7 [15–90]	0.674
Ki67 proliferation index	54.2 ± 16.8 [39.7–67.6]	43.4 ± 20.7 [25.5–57.2]	0.002

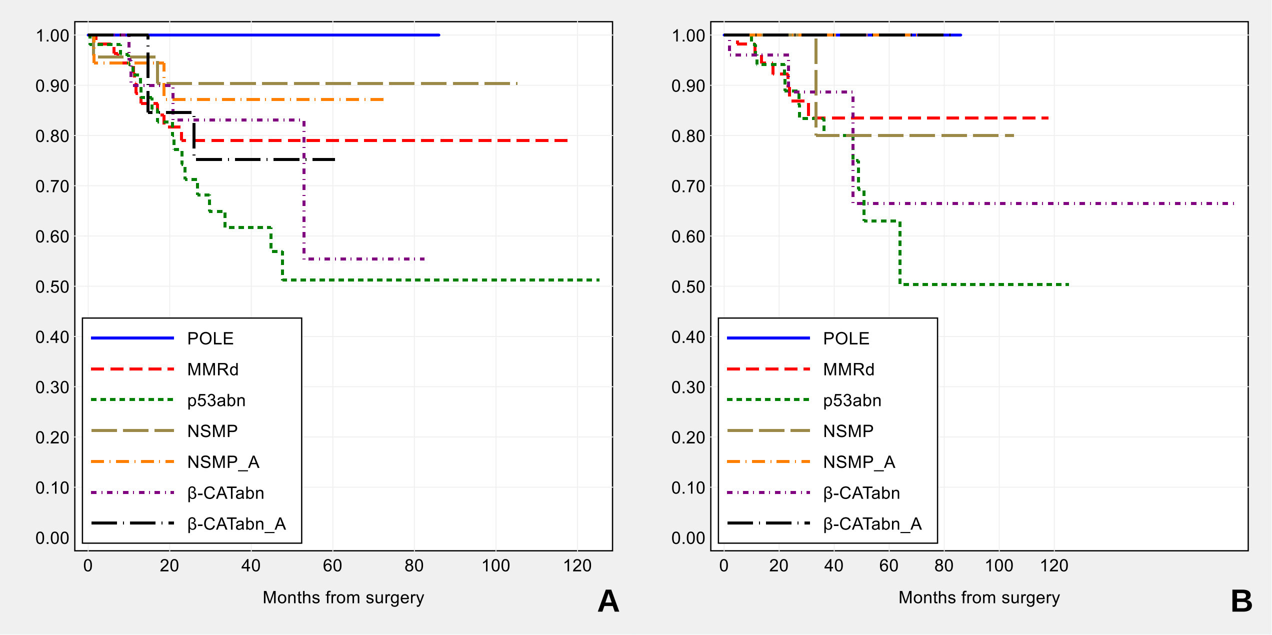

Figure S2. Kaplan–Meier estimates of disease-free survival (A) and overall survival (B) in TCGA case series, by molecular subgroup including β-catenin and ARID1A alterations (A: log-rank $\chi^2 = 11.24$, P-value = 0.081; B: log-rank $\chi^2 = 11.69$, P-value = 0.069).
